# *Dunaliella salina* Attenuates Diabetic Neuropathy Induced by STZ in Rats: Involvement of Thioredoxin

**DOI:** 10.1155/2020/1295492

**Published:** 2020-01-02

**Authors:** Farouk K. El-Baz, Abeer Salama, Rania A. A. Salama

**Affiliations:** ^1^Plant Biochemistry Department, National Research Centre (NRC), 33 El Bohouth St. (Former El-Tahrir St.), 12622 Dokki, Giza, Egypt; ^2^Pharmacology Department, National Research Centre (NRC), 33 El Bohouth St. (Former El-Tahrir St.), 12622 Dokki, Giza, Egypt; ^3^Toxicology and Narcotics Department, National Research Centre (NRC), 33 El Bohouth St. (Former El-Tahrir St.), 12622 Dokki, Giza, Egypt

## Abstract

Diabetic neuropathy (DN) is a widespread disabling disorder including peripheral nerves' damage. The aim of the current study was to estimate the potential ameliorative effect of *Dunaliella salina (D. salina)* on DN and the involvement of the thioredoxin. Diabetes was induced by streptozotocin (STZ; 50 mg/kg; i.p). Glimepiride (0.5 mg/kg) or *D. salina* powder (100 or 200 mg/kg) were given orally, after 2 days of STZ injection for 4 weeks. Glucose, total antioxidant capacity (TAC), superoxide dismutase (SOD), and catalase (CAT) serum levels as well as brain contents of thioredoxin (Trx), tumor necrosis factor-alpha (TNF-*α*), and interleukin-6 (IL-6) were measured with the histopathological study. STZ-induced DN resulted in a significant (*P* < 0.05) rise in glucose blood level and brain contents of TNF-*α* and IL-6 and produced a reduction in serum TAC, SOD, CAT, and brain Trx levels with irregular islets of Langerhans cells and loss of brain Purkinje cells. Treatment with glimepiride or both doses of *D. salina* alleviated these biochemical and histological parameters as compared to the STZ group. *D. salina* has a neurotherapeutic effect against DN via its inhibitory effect on inflammatory mediators and oxidative stress molecules with its upregulation of Trx activity.

## 1. Introduction

Diabetic neuropathy (DN) is the most common diabetes complication and its prevalence ranges from 40% to 50% of patients with diabetes. DN induced foot ulcer pain, disability, and recurrent hospitalizations. There is no treatment for DN other than glycemic control [1]. Epidemiology of DN involves poor glycemic control that is a major risk factor for its development and other factors as hyperlipidemia, hypertension, obesity, cigarette smoking, and consumption of alcohol. Diabetes mellitus is associated with neurodegenerative disorders [2]. Hyperglycemia is considered a central key to DN pathogenesis and induces the formation of reactive oxygen species (ROS) damaging the nerves [3, 4] and provokes sensory symptoms that start in the toes then affect the upper limbs by time which is diagnosed by loss of pain sensation [5] using thermal hot plate test [6]. Glucose is necessary to supply central nervous system with energy [7] and hyperglycemia in diabetes can induce also a variety of complications such as nephropathy, retinopathy, and increased risk of cardiovascular disease [8] that are induced by an injection of streptozotocin (STZ) that selectively destructs insulin-producing *β*-cells of the pancreas experimentally and leads to brain injury [9].

Oxidative stress contributes to diabetes and DN, through the dysfunction of pancreatic *β*-cell, in which *β*-cells express low levels of catalase, glutathione peroxidases, and antioxidant enzymes and slowly detoxify ROS [10]. In the DN process, superoxide (O_2_·^−^) is the most common ROS and induces other ROS as it is converted to hydrogen peroxide (H_2_O_2_) that is detoxified by superoxide dismutase (SOD) and catalase [1]. Another key antioxidant system in DN is thioredoxin (Trx) which is localized in the mitochondria and the cytoplasm, protects cells from oxidative stress through its disulfide reductase activity, and has as a reciprocal role in disease pathogenesis: autoimmune diseases and cancer [11]. Experimental diabetes impaired Trx in the brain [12].

Antioxidant therapy suppressed oxidative stress in DN. *Dunaliella salina* (*D. salina*) is a natural source of carotenoids and is considered as an antioxidant therapy improving diabetes associated with oxidative stress [13]. Antioxidant defense pathway against oxidative stress and inflammation in DN is an essential task within the brain. Therefore, the present study aimed to evaluate the ameliorative efficacy of *D. salina* against oxidative stress and inflammation in DN induced by STZ in rats through the upregulation of Trx.

## 2. Materials and Methods

### 2.1. Cultivation of *D. salina* in the Vertical Photobioreactor

Algal species *D. salina* isolated from a salt pond in Al-Fayoum are grown by using bold media for algal isolation and purification [14]. After growing *D. salina* for 10 days under lab conditions, they are then transferred to a vertical photobioreactor with a capacity of 4000 L. Reservoir (1000 L) tank associated pipe work proprietary in line pigging systems was used for removal of all biofilms. In addition 10 L basket centrifuge for harvesting connected to the system was used. Alga Connect Data Acquisition System was used for online measurements.

Tap water is used for the cultivation of algae in the PBR. Water is sterilized using hypochlorite; after that sodium thiosulphate is added. Chlorine test is done to ensure no residual chlorine is present. A nutrient solution of bold was used for growing *D. salina*. One millilitre of micronutrient solution was added to the culture medium. To ensure the purity of the culture, samples are taken regularly and examined microscopically. The culture is left to grow until the biomass is reached the maximum (2–2.5 gm/L). Algal biomass is harvested using basket centrifuge at 2000 rpm and dried in a sun dryer where the temperature reached approximately 45°C and then grounded into a homogeneous fine powder.

### 2.2. Drugs, Chemicals, and Kits

Glimepiride was obtained from Sanofi-Aventis, Egypt. STZ, diethyl ether, sodium citrate, and formaldehyde were obtained from Sigma Aldrich Chemical Co., USA. Total antioxidant capacity (TAC), Superoxide dismutase (SOD), and catalase were purchased from Biodiagnostic, Egypt. Thioredoxin (Trx), tumor necrosis factor-alpha (TNF-*α*), and interleukin-6 (IL-6) were purchased from NOVA, Beijing, China, Eliza kits.

### 2.3. Animals

Adult male albino Wister rats weighing 150–200 gm were obtained from the animal house at the National Research Centre (Giza, Egypt) and were fed a standard laboratory diet and tap water ad libitum. Experimental animals were housed in an air-conditioned room at 22–25°C with a 12 h light/dark cycle. All animals received human care and the study protocols were carried out according to the ethical guidelines for care and use of experimental animals approved by the Ethical Committee of the National Research Centre.

### 2.4. Experimental Design

DN was induced by a single intraperitoneal injection of STZ (50 mg/kg) dissolved in 0.1 M citrate buffer (pH 4.5) [15, 16]. Fifty adult male albino Wister rats were allowed to drink a 5% glucose solution overnight to overcome the drug-induced hypoglycemia. Blood samples were taken 48 h after injection of STZ to ensure that diabetes has been induced and fasting plasma glucose levels of rats were determined using glucose strips (One Touch SureStep Meter, LifeScan, Calif, USA). Rats with plasma glucose concentration ˃300 mg/dl were considered diabetic and included in the experiment [17]. Rats were assigned randomly into five groups. Group 1: normal control rats were treated with the same volume citrate buffer only without STZ for 30 days. Group 2: diabetic control rats (STZ). Group 3: diabetic rats received glimepiride reference drug (0.5 mg/kg; p.o.) [18] for 30 days. Groups 4 and 5: diabetic rats received *D. salina* powder (100 & 200 mg/kg) [19, 20] for 30 days.

### 2.5. Effects of *D. salina* on Pain Perception (Hot Plate Test)

A hot plate test was conducted using an electronically controlled hot plate (Ugo Basile, Italy) adjusted at 52 ± 0.1°C, and the time elapsed until either paw licking or jumping occurs is recorded [21].

### 2.6. Preparation of Blood Samples and Determination of Serum Levels of TAC, SOD, and Catalase

At the end of the 30 days of treatment, rats were anesthetized with pentobarbital sodium and blood samples were collected for biochemical analyses. Three ml blood was withdrawn from the retro-orbital plexus vein of each rat for biochemical assays. Blood samples were left to clot at room temperature then centrifuged at 1500 rpm for 10 min for serum separation, and serum samples were stored at −20°C in order to determine TAC, SOD, and catalase serum levels.

### 2.7. Preparation of Tissue Homogenate and Determination of Brain Contents of Trx, TNF-*α,* and IL-6

The brains were then excised and washed with saline. Brains were placed in ice-cold phosphate buffer (pH 7.4) to prepare the 20% homogenate that was used for the estimation of brain contents of Trx, TNF-*α,* and IL-6.

Brain contents of Trx, TNF-*α,* and IL-6 were determined using ELISA (Enzyme-Linked Immunosorbent Assay) kit. We followed the manufacturer's instructions of NOVA kit, Beijing, China, for calculating the results. Standards and samples were pipetted into wells with immobilized antibodies specific for rat Trx, TNF-*α,* and IL-6 and then were incubated 30 min at 37°C. After incubation and washing, horseradish peroxidase-conjugated streptavidin was pipetted into the wells and incubated 30 min at 37°C, which was washed once again. Chromogens A & B were added to the wells and incubated 15 min at 37°C; color developed proportionally to the amount of Trx, TNF-*α,* and IL-6 bound. Color development was discontinued (Stop Solution) and after 10 min color intensity was measured at 450 nm.

### 2.8. Histological Examination

The parts of the brain were fixed in 10% formalin solution then dehydrated in ascending grades of alcohol and embedded in paraffin. Four sections/group, at 4 *μ*m thickness, were taken and stained with hematoxylin and eosin (H & E).

### 2.9. Statistical Analysis

All the values are presented as means ± standard error of the means (SE). Data were evaluated by one-way analysis of variance followed by Tukey's multiple comparisons test. Graph pad Prism software, version 5 (Inc., San Diego, USA) was used to carry out these statistical tests. The difference was considered significant when *P* < 0.05.

## 3. Results

### 3.1. Effects of *D. salina* on Pain Perception (Hot Plate Test)

The results indicated, in diabetic rats, a significant loss of pain perception as indicated by elevated withdrawal time in hot plate, while the treatment with *D. salina* powder (100 & 200 mg/kg) reduced the withdrawal time in hot plate as compared to the STZ group. In addition, *D. salina* (200 mg/kg) was more effective by 20% than the standard drug, glimepiride (Table 1).

### 3.2. Effects of *D. salina* on Blood Glucose Levels

**Figure 1:**
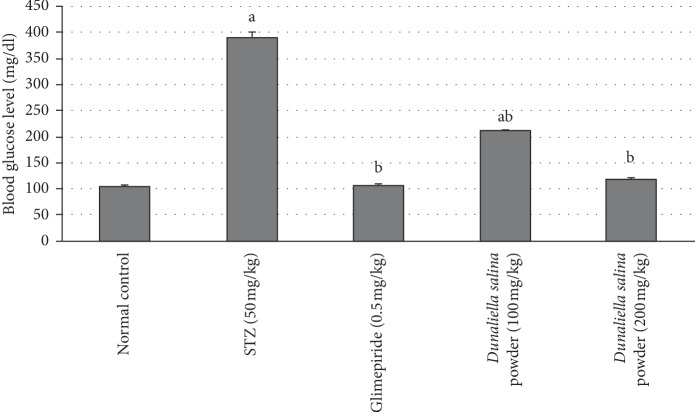
Effects of *D. salina* on blood glucose level. Data are presented as the mean ± S.E. (*n* = 8). Statistical analysis was performed by one-way analysis of variance followed by Tukey's multiple comparisons test. ^a^significant from the normal group. ^b^significant from the STZ group at *P* < 0.05.

Animals injected with STZ exhibited a significant elevation in glucose blood levels after 30 days by 2.7-fold, when compared to normal animals. Treatment with glimepiride reduced glucose blood levels after 30 days by 72% when compared to STZ animals. Treatment with *D. salina* powder (100 & 200 mg/kg) reduced blood glucose levels after 30 days by 46% and 69%, respectively, as compared to the STZ group (Figure 1).

### 3.3. Effects of *D. salina* on Serum Oxidative Stress Biomarkers

A reduction in serum levels of SOD, CAT, and TAC was observed in the STZ group by 45%, 32%, and 51% respectively, as compared to normal control values. Treatment with glimepiride increased serum levels of SOD, CAT, and TAC by 47%, 30%, and 87%, respectively, as compared to the STZ group. Also, treatment with *D. salina* powder (100 mg/kg) revealed an elevation in serum levels of SOD and TAC only by 49%, and 82%, respectively, as compared to the STZ group, while treatment with *D. salina* powder (200 mg/kg) increased serum levels of SOD, CAT, and TAC by 76%, 31%, and 93%, respectively, as compared to the STZ group (Table 2).

### 3.4. Effects of *D. salina* on Brain Content of Trx

**Figure 2:**
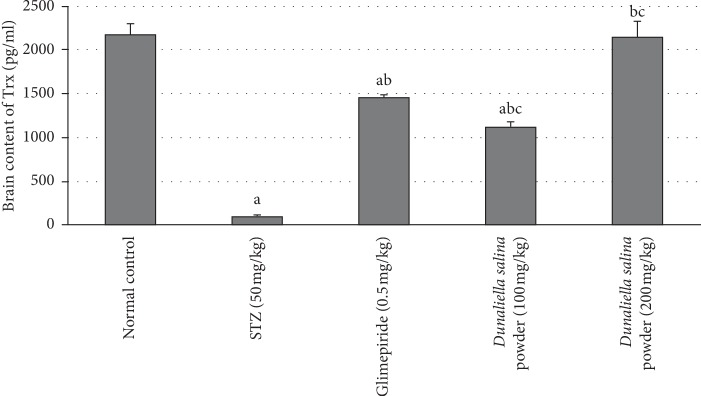
Effects of *D. salina* on brain contents of thioredoxin (Trx). Data are presented as the mean ± S.E. (*n* = 8). Statistical analysis was performed by one-way analysis of variance followed by Tukey's multiple comparisons test. ^a^significant from the normal group. ^b^significant from the STZ group. ^c^significant from glimepride group at *P* < 0.05.

STZ injection, after 4 weeks, reduced a brain content of Trx by 97%, as compared with normal values. Treatment with glimepiride elevated a brain content of Trx by 13-fold as compared to the STZ group. Also, treatment with *D. salina* powder (100 mg/kg) produced a rise in brain content of Trx by 24-fold, treatment with *D. salina* powder (200 mg/kg) increased brain content of Trx by 28-fold as compared to STZ group. In addition, *D. salina* (200 mg/kg) has a higher Trx by 46% than the standard drug glimepiride (Figure 2).

### 3.5. Effects of *D. salina* on Brain Contents of Inflammatory Biomarkers

**Figure 3:**
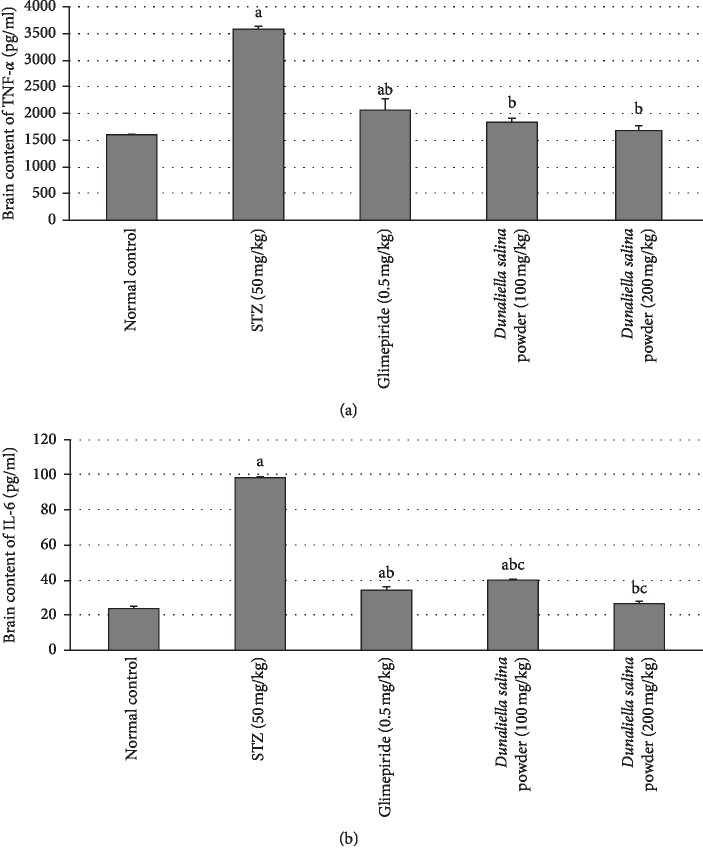
Effects of *D. salina* on brain contents of (a) TNF-*α* and (b) IL-6. Data are presented as the mean ± S.E. (*n* = 8). Statistical analysis was performed by one-way analysis of variance followed by Tukey's multiple comparisons test. ^a^significant from the normal group. ^b^significant from the STZ group. ^c^significant from glimepride group at *P* < 0.05.

Inflammation in the brain was induced by STZ that was evidenced by significant increases in brain contents of TNF-*α* and IL-6 by 1.3-fold and 3.1-fold, respectively, as compared to normal control values. Treatment with glimepiride decreased brain contents of TNF-*α* and IL-6 by 42% and 65%, respectively, as compared to the STZ group. Also, treatment with *D. salina* powder (100 mg/kg) showed a reduction in brain contents of TNF-*α* and IL-6 by 49% and 59%, respectively. Treatment with *D. salina* powder (200 mg/kg) decreased brain contents of TNF-*α* and IL-6 by 53% and 73%, respectively, as compared to STZ group. In addition, *D. salina* (200 mg/kg) decreased IL-6 by 22% as compared with the standard drug glimepiride (Figure 3).

### 3.6. Histopathological Results

**Figure 4:**
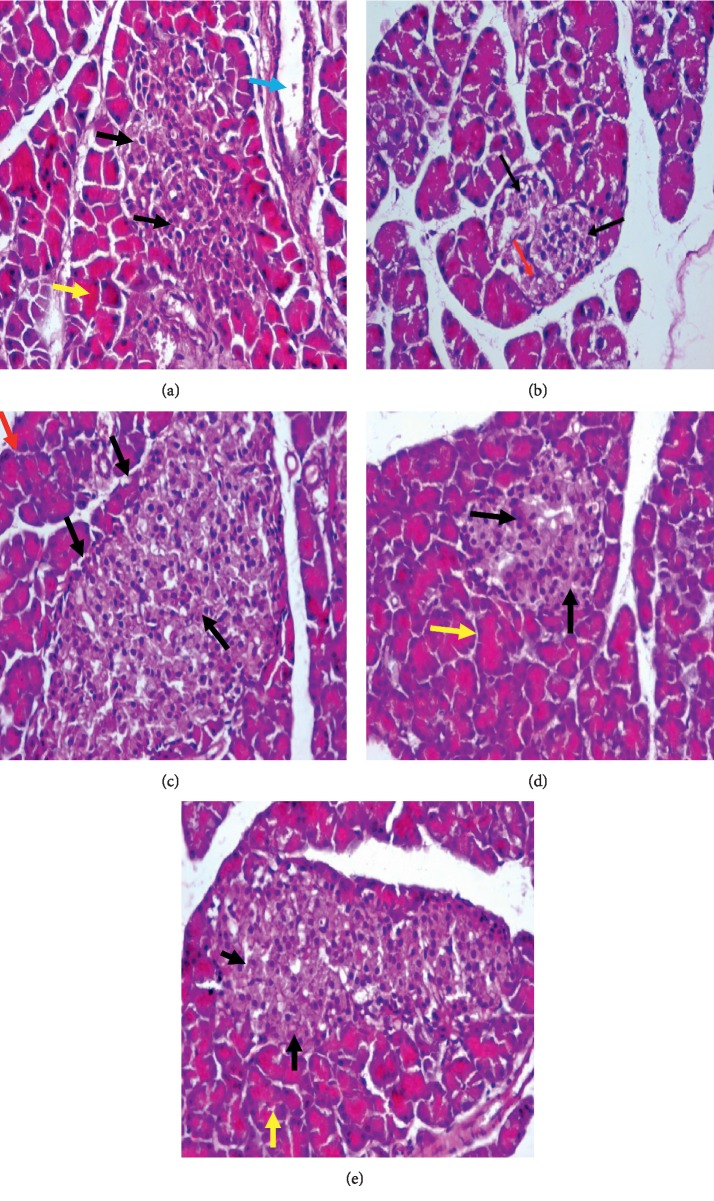
Pancreatic section from the normal control group showed pancreatic islets were shaped regularly and arranged evenly, with normal islets of Langerhans (black arrows) and normal acini tissues (yellow arrows) and duct (blue arrow) (a). Pancreatic section from (STZ) control group showed pancreatic islets with irregular islets of Langerhans cells, not well defined (black arrows), necrosis of cells (red arrow) (b). Pancreatic section from the glimepiride group showed pancreatic islets were shaped almost regularly and arranged evenly, with normal islets of Langerhans (black arrows) and normal acini tissues (red arrows) (c). Pancreatic section from *D. salina* powder 100 mg/kg group showed pancreatic islets were shaped near regularly and arranged evenly, with almost normal islets of Langerhans (black arrows) and normal acini tissues (red arrows) (d). Pancreatic section from *D. salina* powder 200 mg/kg group showed pancreatic islets were shaped almost regularly and arranged evenly, with almost normal islets of Langerhans (black arrows) and normal acini tissues (red arrows) (e). (H&E, x400).

The pancreatic section from the normal control group showed regular and normal islets of Langerhans (black arrows) and normal acini tissues (yellow arrows), and duct (blue arrow) (Figure 4(a)). The pancreatic section from the STZ group showed irregular islets of Langerhans cells (black arrows) and necrosis of cells (red arrow) (B). The pancreatic section from glimepiride showed almost regular and normal islets of Langerhans (black arrows) and normal acini tissues (red arrows) (C). Pancreatic section from *D. salina* powder (100 mg/kg) group showed near regular and almost normal islets of Langerhans (black arrows) and normal acini tissues (red arrows) (D). Pancreatic section from *D. salina* powder (200 mg/kg) group showed almost regularly and almost normal islets of Langerhans (black arrows) and normal acini tissues (red arrows) (E). (H & E, x400).

**Figure 5:**
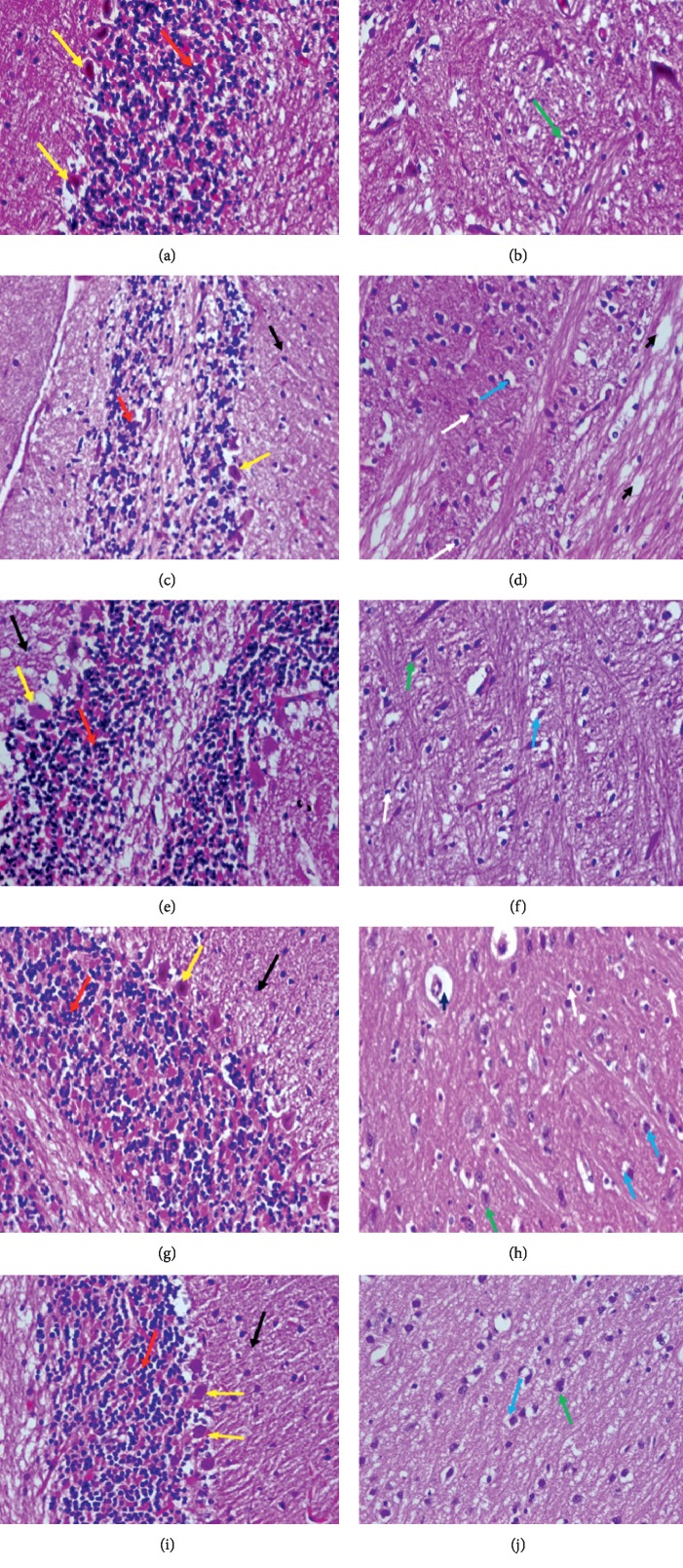
Brain section of normal control group showed the cerebellum showed normal histological features, a well-defined molecular (black arrow), granular (presence of numerous closely packed small cells in the granular layer) (red arrow), and Purkinje layers (large Purkinje cells) (yellow arrow) (a), with normal structure of neuronal cells of the frontal cortex (green arrow) (b). Brain section of the (STZ) control group showed the cerebellum edematous molecular (black arrow), disorganized and thin granular (presence of numerous closely packed small cells in the granular layer) (red arrow), and Purkinje layers showing moderate loss of Purkinje cells (yellow arrow) (c), neuronal cells of the frontal cortex showing perineuronal edema (blue arrow), showing widespread edema (blackhead arrow) and proliferation of oligodendroglia “satellitosis” (white arrow) (d). Brain section of the glimepiride group showed the cerebellum molecular layer (black arrow), granular (presence of numerous closely packed small cells in the granular layer) (red arrow), and loss of some Purkinje cells (yellow arrow) (e), with normal neuronal cells of the frontal cortex (green arrow), perineuronal edema (blue arrow), and few oligodendroglia “satellitosis” (white arrow) (f). Brain section of the *D. Salina* powder 100 mg/kg group showed the cerebellum molecular (black arrow), granular (presence of numerous closely packed small cells in the granular layer) (red arrow), and widespread necrosis and moderate loss of Purkinje cells (yellow arrow) (g), almost normal neuronal cells of the frontal cortex (green arrow), showing perineuronal edema (blue arrow), proliferation of oligodendroglia “satellitosis” (white arrow) (h). Brain section of the *D. Salina* powder 200 mg/kg group showed the cerebellum almost normal molecular layer (black arrow), granular (presence of numerous closely packed small cells in the granular layer) (red arrow), and Purkinje cells (yellow arrow) (i), with almost normal neuronal cells of the frontal cortex (green arrow) and perineuronal edema (blue arrow) (j). (H & E stain, x400).

Brain section of the normal control group showed normal cerebellum histological features, a well-defined molecular (black arrow), granular layer (red arrow) and Purkinje layers (large Purkinje cells) (yellow arrow) (Figure 5(a)), with normal structure of neuronal cells of the frontal cortex (green arrow) (B). Brain section of the (STZ) control group, showed cerebellum edematous molecular layer (black arrow), disorganized and thin granular layer (red arrow), and Purkinje layers showing moderate loss of Purkinje cells (yellow arrow) (C), with neuronal cells of the frontal cortex, perineuronal edema (blue arrow), widespread edema (blackhead arrow), and proliferation of oligodendroglia “satellitosis” (white arrow) (D). Brain section of the glimepiride group showed the cerebellum molecular layer (black arrow), granular layer (red arrow), and loss of some Purkinje cells (yellow arrow) (E), with normal neuronal cells of the frontal cortex (green arrow), perineuronal edema (blue arrow), and few oligodendroglia “satellitosis” (white arrow) (F). Brain section of the *D. salina* powder (100 mg/kg) group showed the cerebellum molecular (black arrow), granular layer (red arrow), widespread necrosis, and moderate loss of Purkinje cells (yellow arrow) (G), with almost normal neuronal cells of the frontal cortex (green arrow), perineuronal edema (blue arrow), and proliferation of oligodendroglia “satellitosis” (white arrow) (H). Brain section of the *D. salina* powder (200 mg/kg) group showed the cerebellum almost normal molecular layer (black arrow), granular layer (red arrow), and Purkinje cells (yellow arrow) (I), with almost normal neuronal cells of the frontal cortex (green arrow) and perineuronal edema (blue arrow) (J). (H & E stain, x400).

## 4. Discussion

Diabetes, in developed countries, is the main reason for neuropathic pain and it is considered the greatest cause of morbidity and mortality in diabetes patients. Diabetic conditions showed a loss of pain perception resulting from neurodegeneration and the development of DN [22]. In the present study, STZ-induced DN. It produced a hyperglycemic effect that was associated with pain perception loss due to nerve damage, while glimepiride or both doses *D. salina* regulated neuropathy progression in STZ-induced diabetic rats that was evidenced by decreasing the time of withdrawal latency that was evaluated by the hot plate method. *D. salina* in high dose decreased the time of withdrawal latency than glimepiride. Moreover, intraperitoneal injection of STZ exerted a necrotic effect on insulin-producing pancreatic *β*-cells leading to hyperglycemia and a significant decrease in insulin secretion within 48 after its injection. These results are associated with irregular islets of Langerhans cells and necrosis of cells in our histopathological study. Several investigations showed that STZ enters the pancreatic *β*-cells by glucose protein-2 transporter and disrupts the balance between antioxidant and oxidant systems damaging the insulin-producing islet *β*-cells and inducing the progression of diabetes [23, 24]. Another study showed STZ produced islet *β*-cells injury, which in turn elevated glucose level and decreased insulin level [25].

The results of the current study indicated that the administration of glimepiride or both doses *D. Salina* significantly alleviated the adverse effects of STZ-induced diabetes. *D. Salina* treatment decreased glucose blood level and increased insulin level with normal islets of Langerhans when compared with rats treated with STZ alone. Previous work showed a decreased level of glucose after *D. salina* administration in STZ diabetic rats [26].

DN progressed in the STZ-treated rats due to the induction of oxidative stress, and this is evidenced by decreased serum levels of TAC, SOD, and catalase and is associated with moderate loss of Purkinje cells and neuronal cells of the frontal cortex showing perineuronal edema. Previous findings showed that STZ enhanced the oxidative stress susceptibility inducing diabetes [23]. Although diabetic complications pathophysiology is multifactorial, animal studies suggest oxidative stress role through elevated levels of ROS that affect many organs [27–30]. In uncontrolled diabetes, oxidative stress is the main feature [31, 32] in which the reduction of antioxidant enzymes activity occurred with elevated blood glucose levels [33]. Moreover, diabetes provoked pathological changes in the central nervous system and damaged mitochondria in the neurons releasing ROS that produced diabetic brain complications [34].

Treatment with glimepiride or *D. saline* ameliorated oxidative stress indices through an elevation in antioxidant contents of TAC, SOD, and catalase and showed normal Purkinje cells and normal neuronal cells of the frontal cortex. These results may be due to *D. saline* high content of carotenoids. Natural products as carotenoids have benefits on diabetes and protect from oxidative damage caused by ROS [35]. Raposo et al. [36] showed the effect of b- carotene on the glutathione regeneration in human trail slowing the complication of diabetes mellitus. Microalgae containing carotenoids have survival mechanisms that scavenge ROS [36]. The previous study exhibited that *D. saline* has an antioxidant effect and recovered the decreased GSH levels in rats [37].

Thioredoxin (Trx) and glutathione are antioxidant thiol-reductase systems that scavenge ROS protecting cells from oxidative stress [38] and regulate redox balance in the brain [39]. Trx is regulated by a thioredoxin-interacting protein (Trxip) [40, 41] which decreases Trx activity inducing oxidative stres and inhibiting cell growth [42]. Trxip links oxidative/glucose stress, inflammation, and cellular injury through a central signaling hub that makes it a promising new target for brain therapy [43]. Trxip decreases the binding of Trx with apoptosis signal-regulating kinase 1 (ASK1) stimulating an ASK1 apoptosis mediated pathway [44]. In this study, hyperglycemia induced by STZ inhibited the thioredoxin antioxidative role, while glimepiride or both doses of *D. saline* treatment elicited upregulation of Trx activity in rat brain compared with the STZ group. Another study indicated that STZ produced its deleterious metabolic effect in diabetes through the downregulation of the thioredoxin activity [45] and enhanced the levels of Trxip mRNA in diabetic rat brains [12]. Trxip expression after middle cerebral artery occlusion is elevated in hyperglycemic-ischemic mice brains with inflammatory mediators release [46]. Oxidative stress upregulated inflammatory molecules, TNF-*α* and IL-6, production [47, 48]. TNF-*α* alters insulin receptor substrate phosphorylation inhibiting the insulin signaling pathway [49]. In the present DN experimental model, STZ injection produced a significant increase in TNF-*α* and Il-6. Hyperglycemia may trigger inflammatory process elevating proinflammatory cytokines, IL-6 and TNF-*α*, expression, possibly due to ROS production [50] or reduction of antioxidant defense systems [51, 52]. Glimepiride or both doses *D. salina* administration after STZ treatment resulted in lower serum levels of IL-6 and TNF-*α* when compared with the diabetic group*. D. saline* administration recovered increased TNF-*α* and IL-6 levels in previous work [53]. Finally, *D. saline* treatment in high dose level has higher Trx and lower IL-6 than the standard drug glimepiride.

## 5. Conclusion

*D. salina* ameliorates DN through stimulation of the two thiol-reductase antioxidant systems, GSH and Trx, and other antioxidant enzymes and inhibition of inflammatory molecules, so it protects the neurons of the brain from oxidative stress and inflammation induced by STZ in rats.

## Figures and Tables

**Table 1:** Effects of *D. salina* on pain perception (hot plate test).

	Normal control	STZ (50 mg/kg)	Glimepiride (0.5 mg/kg)	*Dunaliella salina* powder (100 mg/kg)	*Dunaliella salina powder* (200 mg/kg)
Withdrawal time (sec)	16.20 ± 0.51	56.80 ± 1.71^a^	31.00 ± 0.45^ab^	32.50 ± 1.12^ab^	24.80 ± 1.06^abc^

Data are presented as the mean ± S.E. (*n* = 8). Statistical analysis was performed by one-way analysis of variance followed by Tukey's multiple comparisons test. ^a^significant from the normal group. ^b^significant from the STZ group. ^c^significant from glimepiride group at *P* < 0.05.

**Table 2:** Effects of *D. salina* on serum oxidative stress biomarkers.

	Normal control	STZ (50 mg/kg)	Glimepiride (0.5 mg/kg)	*Dunaliella salina* powder (100 mg/kg)	*Dunaliella salina powder* (200 mg/kg)
TAC (mM/L)	0.62 ± 0.04	0.30 ± 0.05^a^	0.57 ± 0.03^b^	0.55 ± 0.07^b^	0.59 ± 0.03^b^
SOD (U/ml)	334.13 ± 5.45	182.66 ± 12.68^a^	268.65 ± 11.96^b^	272.90 ± 22.44^b^	320.65 ± 29.71^b^
Catalase (U/L)	618 ± 3.99	421.31 ± 25.01^a^	546.66 ± 16.79^b^	497.33 ± 1.63^a^	553 ± 33.48^b^

Data are presented as the mean ±  S.E. (n=8). Statistical analysis was performed by one-way analysis of variance followed by Tukey's multiple comparisons test. ^a^significant from the normal group. ^b^significant from the STZ group at *P* < 0.05.

## Data Availability

The data used to support the findings of this study are available upon request to the corresponding author.
